# Solubility and Thermal Degradation of Quercetin in CO_2_-Expanded Liquids

**DOI:** 10.3390/molecules25235582

**Published:** 2020-11-27

**Authors:** Larissa P. Cunico, Andrés Medina Cobo, Said Al-Hamimi, Charlotta Turner

**Affiliations:** 1 Centre for Analysis and Synthesis (CAS), Department of Chemistry, Lund University, P.O. Box 124, SE-221 00 Lund, Sweden; larissa.cunico@chem.lu.se (L.P.C.); amcobo@ucm.es (A.M.C.); said.alhamimi@oq.com (S.A.-H.); 2 OQ, SablaX, P.O Box 261, P.C 118 Muscat, Oman

**Keywords:** gas-expanded liquid, green solvent, quercetin, solubility, subcritical fluid, thermal degradation

## Abstract

The solubility of quercetin and its thermal degradation was studied in CO_2_-expanded ethanol and ethyl lactate. An equipment setup was constructed that enabled the separation of the products of degradation while quantifying the solubility of quercetin. Three different conditions of temperature were analyzed (308, 323, and 343 K) at 10 MPa. Higher solubility and thermal degradation of quercetin were observed for CO_2_-expanded ethyl lactate in comparison with CO_2_-expanded ethanol. At the same time, as the amount of CO_2_ was increased in the CO_2_-expanded liquids mixtures, the thermal degradation of quercetin decreased for almost all the conditions of temperature considered in this work. The importance of considering thermal degradation while performing solubility measurements of compounds that are thermally unstable such as quercetin was highlighted.

## 1. Introduction

Quercetin is one of the most abundant flavonoids observed in many types of fruits, vegetables, leaves, and grains. The interest in quercetin has risen in the past years due to its medicinal benefits, such as anti-inflammatory, antioxidant, and anti-carcinogenic activity [1,2]. Quercetin can be extracted from natural matrices, and different techniques for the extraction can be found in literature, such as solid-liquid extraction [3], and pressurized liquid extraction [4], among others. However, extraction is generally limited by solubility, mass transfer resistance, and partitioning [5]. Quercetin presents low stability, suffering from rapid hydrolysis in aqueous solution [6], being thermally unstable [7], and unstable in the presence of oxygen [8] and light [9]. Therefore, it is important to use a method for the extraction of quercetin that provides high solubility in the solvent and that favors fast mass transfer and partitioning, resulting in quicker extraction with low residence time and thereby less risk of degradation.

The solubility of quercetin has been studied before in different solvents, such as subcritical water, water + methanol, water + ethanol, acetonitrile, acetone, *tert*-amyl alcohol, and carbon dioxide (CO_2_) + ethanol as a modifier, and in neat CO_2_ [10,11,12,13]. The available data of solubility of quercetin in the literature show disagreement between the authors, as observed by Abraham and Acree Jr. [14] for the solubility of quercetin in water. Moreover, it is known that quercetin is relatively polar [15], and in this case solvents with moderate to high polarity are a better option for the solubility of quercetin [16].

Carbon dioxide expanded liquids (CXLs) are solvents containing higher amounts of organic solvent in mixtures with CO_2_, typically 0.5 molar fraction or higher, and they present the benefits of fine-tuning physicochemical properties combined with high mass transfer, mediated by the presence of CO_2_. CXLs have for instance been used as a solvent or anti-solvent in catalytic reactions [17] and in particle formation processes [18]. Lately, it has also become more commonly used as a solvent in extractions [19], most likely due to the high diffusivity of the solvent in the sample, which increases the extraction rate in comparison to using only an organic solvent [20]. In our previous work [21], CO_2_-expanded ethanol was used for the solubility study of curcumin, a polyphenol present in turmeric (*Curcuma longa L.*). This study showed higher solubility in higher proportions of organic solvents (50 to 90% mol) in comparison with the use of CO_2_ with less co-solvent (5% mol). Another benefit of CXLs is the typical use of low to moderate temperatures, which prevents the thermal degradation of compounds such as quercetin, which thanks to the added CO_2_ will still keep a low viscosity of the solvent.

In this work, CO_2_-expanded ethanol and CO_2_-expanded ethyl lactate were used for the first time for the solubility measurements of quercetin. Ethanol and ethyl lactate were selected because they are considered green solvents with applications in the food and pharmaceutical industries [22], and because of their difference in polarity and dielectric properties (dipole moment is 1.69 D and 3.46 D for ethanol and ethyl lactate, respectively, while the dielectric constant at 298 K is 24 and 16 for ethanol and ethyl lactate, respectively). Different amounts of CO_2_ (10, 30, and 50% mol) and temperatures (308, 323, and 343 K) were studied aiming to fine tune the physicochemical properties of the solvents. Only one pressure (10 MPa) was applied, since liquids containing less than 50% mole of CO_2_ are not as compressible as supercritical fluids, hence, pressure is of less importance than molar composition and temperature. Due to the low stability of quercetin, its thermal degradation was also investigated in the same conditions of temperature and pressure used for the solubility measurements. The equipment setup used for the solubility and thermal degradation allows the separation of quercetin from its products of degradation, which is important for the correct quantification of the quercetin. Another advantage of the equipment setup used is the on-line analysis of quercetin concentration, which can also prevent any degradation by contact with light or oxygen, which inherently occurs in off-line analysis.

## 2. Results and Discussion

### 2.1. Thermal Degradation Measurements

The two forms of quercetin considered in this work, dihydrate and anhydrous forms, presented different types of dispersions in the CXLs when performing measurements at or over the saturation concentrations. The observed time for the excess quercetin to precipitate and settle was longer than for other compounds, e.g., curcumin, used previously in the same equipment [21]. However, after approximately one hour, the excess anhydrous quercetin had totally precipitated and settled, which allowed the analysis of the amount that was soluble in the CXLs. For dihydrate quercetin, after approximately two hours, particles in dispersion were still observed in the CXLs. Because of this long time, which could cause unnecessary increment of thermal degradation due to the longer exposure to elevated temperatures, all the solubility and thermal degradation measurements were performed using only anhydrous quercetin. A similar experience was also observed by Srinivas et al. [10] for the solubility measurements of quercetin in subcritical water, where the crystals of dihydrate quercetin seemed to aggregate, and showed unstable solubility measurements and solvent flow.

**Figure 1:**
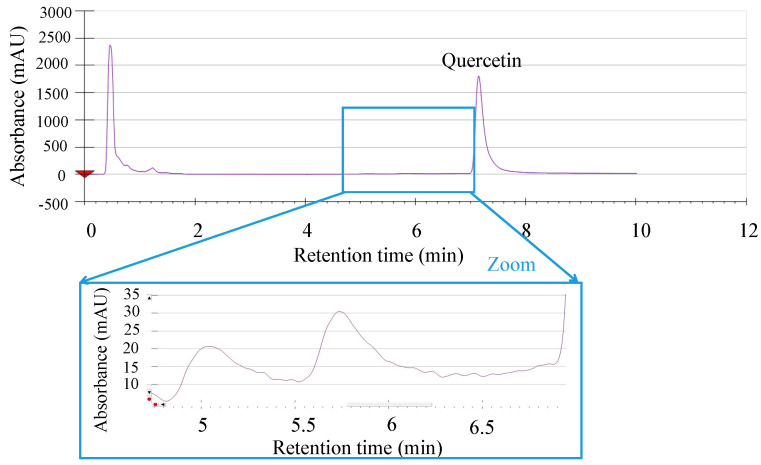
A supercritical fluid chromatography separation method with diode array detection (SFC-DAD) chromatogram of the separation of quercetin (eluting at approximately 7 min) from two other compounds detected that could be possible products of degradation (in zoom). SFC-DAD method: Backpressure 12 MPa, temperature 323 K, flow rate 3 mL/min, gradient from 10 to 35 volume % of ethanol in 8 min and a holding time of 2 min, DAD wavelength 200 to 600 nm with quercetin quantification at 369 nm. For more information, see Section 3.3.

A supercritical fluid chromatography separation method with diode array detection (SFC-DAD) was developed to enable the quantification of quercetin without the interference of potential degradation products. The SFC-DAD method demonstrated the separation of quercetin from two other peaks that could be possible products of degradation (see Figure 1). This analysis method was used for all solubility and degradation rate estimations.

**Figure 2:**
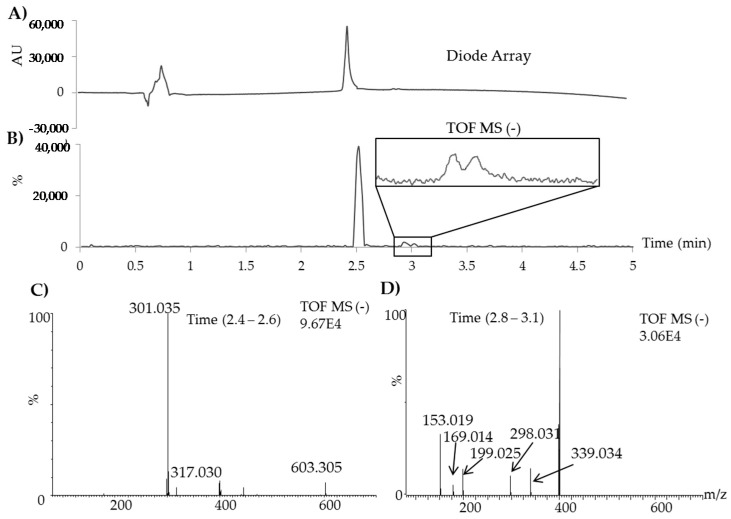
An ultrahigh performance liquid chromatography method with quadrupole time-of-flight detection (UHPLC-DAD-QTOF/MS) analysis of a treated sample of quercetin: (**A**) DAD chromatogram at 365 nm, (**B**) total ion current chromatogram in negative mode, (**C**) spectrum for the main peak, quercetin with some partly co-eluting degradation products, and (**D**) the spectrum for the other two peaks as degradation products of quercetin. The mass of *m/z* 400 is a background contamination, having also been detected in the blank injection solvent.

Furthermore, an ultrahigh performance liquid chromatography method with quadrupole time-of-flight detection (UHPLC-DAD-QTOF/MS) was developed and used to identify the degradation products of quercetin. Because of reversed polarities of mobile phase and stationary phase in UHPLC compared to SFC, the elution order of the peaks in UHPLC is reversed (Figure 2A,B). According to the obtained QTOF/MS results several masses (*m/z*) could be tentatively assigned to likely degradation products of quercetin. The extracted spectrum for the peak(s) eluting between 2.4 and 2.6 min (Figure 2C) indicates presence of quercetin (*m/z* 301.053, t_R_ 2.52 min), also showing traces of dimer quercetin molecules (*m/z* 603.305), and likely also some co-eluting oxidation products such as quercetin quinone (*m/z* 299.019, t_R_ 2.57 min), observed by [23,24,25] and 2-(3,4-dihydroxybenzoyl)-2,4,6-trihydroxy-benzofuran-3-one *(m/z* 317.030, t_R_ 2.59 min), also observed in [8]. The masses (*m/z*) for the two other peaks eluting after quercetin (see zoom-in in Figure 2B, as well as the peaks eluting before quercetin in Figure 1) were extracted together as shown in the spectrum in Figure 2D. The main masses suggested as degradation products of quercetin are 2,4,6-Trihydroxymandelate (m/z 199.025, t_R_ 2.86 min), observed by [9,23,24,26], 3,4-Dihydroxybenzoate (*m/z* 153.019, t_R_ 2.98 min), and 2,4,6-Trihydroxybenzoate (*m/z* 169.014, t_R_ 3.02 min), also observed in references [9,26].

**Figure 3:**
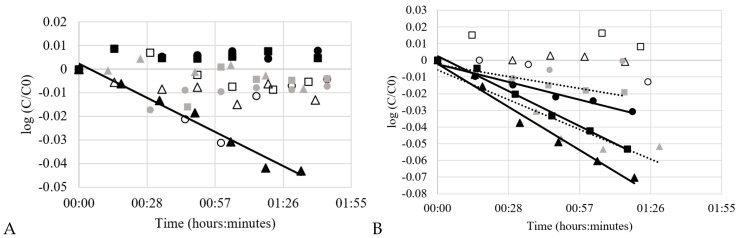
Thermal degradation kinetics of quercetin in: (**A**) CO_2_-expanded ethanol and (**B**) CO_2_-expanded ethyl lactate. For 308 K: ∆ 10% mol CO_2_, □ 30% mol CO_2_, and ○ 50% mol CO_2_. For 323 K: ▲ 10% mol CO_2_, ■ 30% mol CO_2_ and ● 50% mol CO_2_. For 343 K: ▲ 10% mol CO_2_, ■ 30% mol CO_2_, and ● 50% mol CO_2_. Mean values, *n* = 3.

The results obtained for the thermal degradation kinetics study can be seen in Figure 3A for quercetin in CO_2_-expanded ethanol and Figure 3B for quercetin in CO_2_-expanded ethyl lactate. For mixtures with ethanol, thermal degradation was only observed when 10% mol of CO_2_ was added in ethanol at the highest temperature considered in this work (343 K). See trend line in Figure 3A. For mixtures with ethyl lactate, thermal degradation was observed for 10% mol and 30% mol of CO_2_ in ethyl lactate at 323 K and for all concentrations of CO_2_ at 343 K (see trend lines in Figure 3B). The standard deviation of measured concentrations of quercetin (as molar fraction) was 0.09 for 308 K, 0.05 for 323 K, and 0.03 for 343 K in mixtures of CO_2_-expanded ethanol, and 0.50 for 308 K, 0.13 for 323 K, and 0.14 for 343 K in mixtures of CO_2_-expanded ethyl lactate.

The degradation kinetics of quercetin in CXLs seen in Figure 3 follow the first order kinetics. The first order kinetics constant (*k*) can be calculated according to the follow equation:

Log(*C*/*C*_0_) = −*k* × *t* (1)

where *C* is the concentration at the determined time *t* and *C*_0_ is the initial concentration. The first order kinetics constant (*k*) obtained from the experimental data can be seen in Table 1. Higher CO_2_ content seems to help to prevent thermal degradation in the studied cases. The presence of CO_2_ shows slower kinetics of thermal degradation than observed values in the literature for other solvents [27,28]. This can most likely be explained by the dilution caused by CO_2_ in the mixture, thereby lowering the dielectric constant and polarizability of the solvent. As an example, the thermal degradation kinetics constant (*k*) was observed by Wang and Zhao [27] for quercetin to be equal to 4.68 × 10^−4^ min^−1^ at 310 K and 4.08 × 10^−3^ min^−1^ at 323 K in ethanol solution. Relatively severe oxidation of quercetin has also been observed before in literature for alcoholic solutions [9]. Liu et al. [28] showed that the thermal degradation kinetics constant (*k*) for quercetin-3,4′-diglucoside was 7 × 10^−3^ min^−1^ at 383 K in a mixture of water, ethanol, and formic acid (94:5:1, *v*/*v*/*v*). Moreover, the presence of water also showed oxidation of quercetin, as observed before by Buchner et al. [24]. Hence, one explanation for the more obvious degradation in CO_2_-expanded ethyl lactate as compared to in CO_2_-expanded ethanol could be that the water concentration is slightly higher in the former, which could trigger both oxidation and hydrolysis reactions.

### 2.2. Solubility Measurements

Isobaric solubility data was obtained for quercetin in CO_2_-expanded ethanol and CO_2_-expanded ethyl lactate at 10 MPa and temperatures of 308, 323, and 343 K. The results are given in Table 2. Clearly, the lowest concentration of CO_2_ in the CXL gives the highest solubility of quercetin, which is due to quercetin being a relatively polar molecule. It is possible to observe a slight increase of solubility of quercetin in both CO_2_-expanded ethanol and CO_2_-expanded ethyl lactate with increment of temperature. However, in some cases, when relatively severe thermal degradation was observed, such as for ethyl lactate (see Figure 3B), the increment of temperature affected the trueness of the measurements of solubility. Moreover, when the smallest amount of CO_2_ (10% mol) was added to the organic solvent (ethanol or ethyl lactate), higher thermal degradation (check Section 2.1 on thermal degradation measurements) and larger effects on the solubility measurements (Table 2) were observed. When thermal degradation is taken into account in the calculated values of solubility, it is clear how large the bias is due to degradation. This shows the importance of the consideration of degradation in solubility measurements of thermally unstable compounds such as quercetin.

When data is available in literature, such as for the temperature equal to 308 K, the results obtained in this work seem to be in agreement with the value of solubility of quercetin in pure ethanol [11]. Quercetin presents higher solubility in CO_2_-expanded ethyl lactate in comparison with the measurements performed for CO_2_-expanded ethanol. This could be explained by the higher polarity of ethyl lactate [29]. However, a more complete scenario with more experimental data for Kamlet-Taft parameters (polarity, acidity, and basicity are estimated separately) for the CXLs mixtures as well as the solubility of quercetin is needed to try to understand the effect of polarity and hydrogen bonds of the solvent on the solubility of quercetin.

## 3. Materials and Methods

### 3.1. Chemicals

Ethanol (CAS no. 64-17-5) with purity of ≥99.7% was purchased from Solveco—Rosersberg, Sweden, ethyl lactate (CAS no. 687-47-8) with purity of ≥99.0% was purchased from Alfa Aesar—Kandel, Germany, and CO_2_ (CAS no. 124-38-9) with purity of type 5.3 (≥99.9993%) was purchased from AGA— Solna, Sweden. Quercetin dihydrate (CAS no. 6151-25-3) with purity of ≥98.0% was purchased from Fluka—Munich, Germany. Quercetin anhydrous (CAS no. 117-39-5) with purity of ≥95.0% was purchased from Sigma Aldrich—Munich, Germany.

### 3.2. Equipment Setup

The equipment setup used in this work was described in detail in our previous work [21] and the scheme can be found in the Supplementary Material, Figure S1. It contained a variable volume view cell with a sapphire window, where quercetin and the solvent (CXLs) were placed for the equilibration of the set temperature and pressure. CO_2_ was delivered to the variable volume view cell using a piston pump (model 260D, from Teledyne Isco—Lincoln, NE, USA). Stirring was provided by using a stir bar placed inside the view cell and in contact with a magnetic stirrer (model Cimarec I Micro, from Thermo Scientific, Waltham, MA, USAA six-port injection valve containing an injection loop of 1.1 μL was used to connect the variable volume view cell through a recirculation line to a semi-preparative supercritical fluid chromatography (SFC) system from Waters (Thar Investigator—Orlando, FL, USA), containing a photodiode array detector (DAD) from Waters—Orlando, FL, USA. The SFC-DAD was used for the analysis of the amount of quercetin soluble in the CXLs. When the compounds presented high molar absorptivity, the injection valve allowed smaller amounts of the mixture present in the injection loop to be analyzed, which assured that the measurements were within the linear range of Beer–Lambert’s law.

### 3.3. Thermal Degradation Measurements

For the quantification of the thermal degradation rate of quercetin in CO_2_-expanded ethanol and CO_2_-expanded ethyl lactate, continuous measurements of a dissolved amount of quercetin in the CXLs took place for 1.5 h. This period of dissolution and equilibration time was initially investigated by analyzing samples beyond 1.5 h. This time period of 1.5 h allowed the temperature to stabilize at the setpoint, ensured that the concentration of quercetin in the CXL was not changing with time, and that excess quercetin precipitated and settled to the bottom of the view cell. Further, to analyze if the fact of taking measurements was causing any interference to the results, an investigation was performed in which no measurements were taken until 1.5 h, when finally one measurement was taken. The outcome was that sampling during dissolution did not affect the results. To avoid degradation of quercetin before the analysis, quercetin was placed in the variable volume view cell in a dark room, and ethanol respectively ethyl lactate was degassed before use. Because the products of thermal degradation of quercetin could cause interference in the quantification of quercetin using SFC-DAD, a method was developed to separate quercetin from its products of degradation. SFC-DAD was coupled on-line to the view cell, using a mobile phase as similar to the CO_2_-expanded liquids as possible in order to minimize risk for precipitation and losses. The column used for the separation was an Inertsil Diol 150 × 2.1 mm, 5 μm, from GL Sciences (Tokyo, Japan). A mixture of CO_2_ and ethanol was used as mobile phase in the SFC to avoid any contamination to the recirculation line with other organic solvents through the injection valve. The gradient went from 10 to 35 volume % of ethanol in 8 min with a holding time of 2 min (10 min in total). The backpressure was 12 MPa, the temperature was 323 K, and the flow rate was 3 mL/min. For each analysis, 1.1 μL was injected using an in-line coupled injection valve. Quercetin was quantified using a wavelength of 369 nm.

Experiments were conducted at all combinations of temperature (308, 323, and 343 K) and CO_2_ content (10, 30, and 50% mol), and with two co-solvents (ethanol and ethyl lactate). Pressure was constant at 10 MPa. All experiments were conducted in triplicate.

### 3.4. UHPLC-QTOF/MS Analysis

Before the method development for the SFC-DAD, the information about the possible products of degradation of quercetin was considered from literature [8,9,23,24,25,26,30] and an ultrahigh performance liquid chromatography method with quadrupole time-of-flight detection (UHPLC-DAD-QTOF/MS) analysis was performed for the amount collected from the variable volume view cell after 1.5 h. This was done to identify if the number of potential degradation compounds.

A 3 μL sample was injected onto a Waters Acquity UPLC BEH-C18 column (100 mm × 2.1 mm, 1.7 μm; Waters Corporation, Milford, MA, USA) using an ACQUITY UPLC system (Waters Corporation, Milford, MA, USA). The mobile phase consisted of (A) water and (B) methanol, both containing 0.1% (*v/v*) formic acid. The column temperature was 323 K and the flow rate 400 μL/min. Quercetin and its degradation products were eluted using a gradient starting at 15% B, then increasing from 15 to 90% B over 0 to 5 min, with the composition being held at 90% B for 1 min, and finally returned to initial conditions, for 2.5 min. Mass spectrometry detection was performed on a Xevo™ G2 QTof (Waters MS Technologies, Manchester, UK). The mass spectrometer was scanning from 100 to 600 *m/z*, the cone voltage was set to 30 V and the capillary voltage to 2.5 in negative electrospray ionization (ESI) mode. The desolvation gas flow rate was 600 L/h at a temperature of 673 K and the cone gas flow rate was 40 L/h. The source temperature was 393 K. An extracted ion chromatogram (EIC) for suspected *m/z* ions was used to determine the presence of possible products resulted from degradation of quercetin.

### 3.5. Solubility Measurements

For the solubility measurements, calibration curves were established by using known different small amounts of quercetin (between 1.3 mg/mL and 20 mg/mL) in CXLs to assure complete solubility, and the experiments were performed in the same conditions of temperature, pressure, and composition of CO_2_ used for the thermal degradation measurements. An example of a calibration curve can be seen in the Supplementary Materials Figure S2. Larger amounts of quercetin were used for the saturation point, where no additional amount of quercetin could be solubilized in the CXLs. The measurements were performed at the wavelength with maximum absorbance (369 nm) using the hyphenated SFC-DAD method as described above (Section 3.3). All measurements were done in triplicate.

Because thermal degradation was observed in some cases, the method developed for the separation of quercetin from its products of degradation was also used during the analysis of the concentration of the amount of quercetin soluble in CXLs using SFC-DAD.

## 4. Conclusions

Higher solubility and thermal degradation of quercetin were observed for CO_2_-expanded ethyl lactate in comparison with CO_2_-expanded ethanol at the studied conditions of temperature and pressure in this work. For CO_2_-expanded ethanol, the maximum solubility of quercetin was obtained at the highest temperature considered (343 K) and for 30% mol CO_2_ in the mixture. For CO_2_-expanded ethyl lactate, the maximum solubility was observed for the lowest temperature considered in this work (308 K) and for 10% mol CO_2_ in the mixture. The effect of thermal degradation during the solubility measurements of quercetin in CXLs was observed for 323 and 343 K. This highlights the importance of considering thermal degradation and separation of products of degradation while performing solubility measurements of quercetin. If degradation rate constants can be experimentally determined, like what has been done in this study, then solubility can be determined with less bias. In almost all the studied cases, lower thermal degradation is observed by increasing the amount of CO_2_ in the CXLs mixtures. Lower thermal degradation was obtained for quercetin in CXLs than observed before in literature [27,28] for other solvents. This could indicate that CXLs are interesting options of solvents to be considered in extraction and analytical methods of thermally unstable compounds such as quercetin.

## Figures and Tables

**Table 1:** Effect of temperature and CO_2_ concentration on the first order kinetics constant (*k*) for the thermal degradation of quercetin in carbon dioxide expanded liquids (CXLs). “Not observed” means that there was no detected change in quercetin concentration, i.e., no degradation. *n* = 3.

CO_2_ (% mol)	Temperature (K)	*k* (min ^−1^) 10^−4^	
		CO_2_-Expanded Ethanol	CO_2_-Expanded Ethyl Lactate
10	308	Not observed	Not observed
30		Not observed	Not observed
50		Not observed	Not observed
10	323	0.44	7.05
30		Not observed	2.94
50		Not observed	Not observed
10	343	4.83	9.33
30		Not observed	6.83
50		Not observed	4.09

**Table 2:** Quercetin solubility in CO_2_-expanded ethanol and CO_2_-expanded ethyl lactate at 10 MPa. Solubility was also calculated using the first order kinetics constant when thermal degradation was observed (calculated values within parentheses). *n* = 3.

CO_2_ (% mol)	Temperature (K)	Solubility (Molar Fraction) 10^−4^	
		CO_2_-Expanded Ethanol	CO_2_-Expanded Ethyl Lactate
10	308	10.61 ± 0.16	43.22 ± 0.16
30		7.74 ± 0.06	19.24 ± 0.07
50		5.53 ± 0.06	8.93 ± 0.12
10	323	10.67 ± 0.69 (calc. 10.80)	41.71 ± 0.12 (50.68)
30		13.07 ± 0.09	27.74 ± 0.09 (30.09)
50		6.13 ± 0.08	11.16 ± 0.02
10	343	11.87 ± 0.50 (calc. 13.56)	37.49 ± 0.06 (48.51)
30		14.00 ± 0.15	21.98 ± 0.26 (26.55)
50		8.06 ± 0.03	10.18 ± 0.01 (11.40)

## Supplementary Materials

The following are available online, Figure S1: Scheme of the equipment used, Figure S2: Example of a calibration curve (here: quercetin in CO_2_-expanded ethyl lactate, at 343 K, 10 MPa, containing 10%mol of CO_2_).
