# Supplementary material for: Solubility and Thermal Degradation of Quercetin in CO2-Expanded Liquids
Source: Molecules. 2020 Nov 27;25(23):5582. doi: 10.3390/molecules25235582 (PMC7730818; doi:10.3390/molecules25235582)
Supplement: Supplementary file 1 [file molecules-25-05582-s001.pdf]

Supplementary Material

Solubility and thermal degradation of quercetin in CO_2-expanded liquids

Larissa P. Cunico[1], Andrés Medina Cobo[1], Said Al-Hamimi[1,2], Charlotta Turner[1]

1 Lund University, Department of Chemistry, Centre for Analysis and Synthesis (CAS), P.O. Box 124, SE-221 00 Lund, Sweden; larissa.cunico@chem.lu.se; amcobo@ucm.es; said.alhamimi@oq.com.
2 OQ, SablaX, P.O box 261, P.C 118, Muscat, Oman
* Correspondence: charlotta.turner@chem.lu.se (Charlotta Turner), Tel.: +46 46 222 8125.

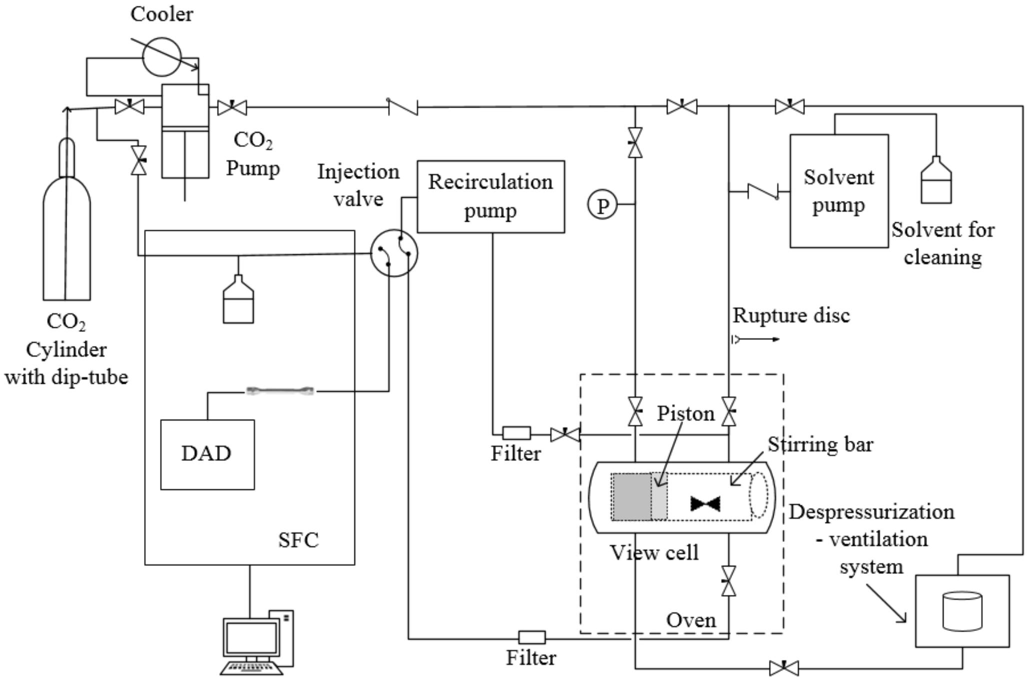

Figure S1. Scheme of the equipment used. For a detailed description on how to operate the system, please see our previous study [Cunico, L. P. et al. Experimental measurements and modeling of curcumin solubility in CO_2-expanded ethanol. J. Supercrit. Fluids 2017, 130, 381-388. Open access: https://doi.org/10.1016/j.supflu.2017.06.018].

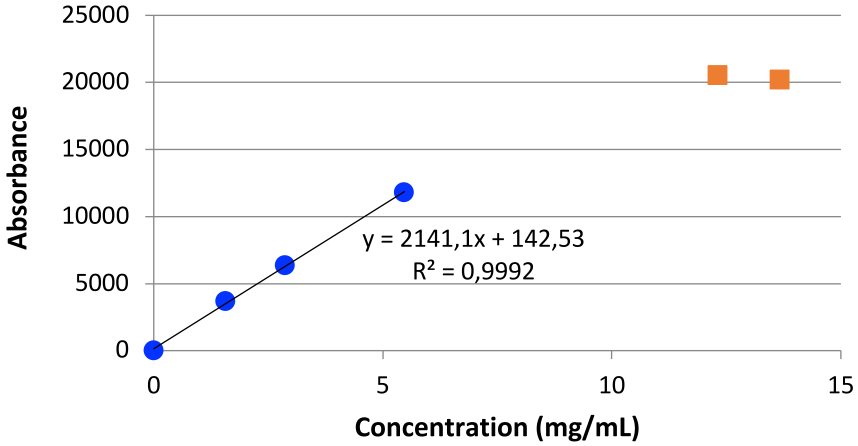

Figure S2. Example of a calibration curve (here: quercetin in CO_2-expanded ethyl lactate, at 343 K, 10 MPa, containing 10 %mol of CO_2). Blue-filled circles mark the calibration points, while the orange-filled squares mark the plateau (horizontal) values giving the solubility of quercetin in the solvent.
